# Graphitisation of Waste Carbon Powder with Femtosecond Laser Annealing

**DOI:** 10.3390/mi13010120

**Published:** 2022-01-12

**Authors:** Lucas Lum, Chong-Wei Tan, Chun Fei Siah, Kun Liang, Beng Kang Tay

**Affiliations:** 1 Centre for Micro- and Nano-Electronics (CMNE), School of Electrical and Electronic Engineering, Nanyang Technological University, 50 Nanyang Ave, Singapore 639798, Singapore; e190013@ntu.edu.sg (L.L.); ChongWei@ntu.edu.sg (C.-W.T.); chunfei001@e.ntu.edu.sg (C.F.S.); liangkun@ntu.edu.sg (K.L.); 2 CNRS-NTU-THALES Research Alliances/UMI 3288, Research Techno Plaza, 50 Nanyang Ave, Singapore 637553, Singapore

**Keywords:** carbon black, femtosecond laser annealing, ablation, nano-crystallisation

## Abstract

Graphitisation of structural characteristics and improvement in electrical conductivity was reported onto waste carbon powder through femtosecond laser annealing. Raman spectroscopy on the carbon powder pre- and post-annealing showed a shift from amorphous-like carbon to graphitic-like carbon, which can be explained by the three-stage model. Electrical I-V probing of the samples revealed an increase in conductivity by up to 90%. An increase in incident laser power was found to be correlated to an increase in conductivity. An average incident laser power of 0.104 W or less showed little to no change in electrical characteristics, while an average incident laser power of greater than 1.626 W had a destructive effect on the carbon powder, shown through the reduction in powder. The most significant improvement in electrical conductivity has been observed at laser powers ranging from 0.526 to 1.286 W. To conclude, the graphitisation of waste carbon powder is possible using post-process femtosecond laser annealing to alter its electrical conductivity for future applications.

## 1. Introduction

Human-generated waste is a key source of pollution and a significant source of environmental damage. Therefore, significant effort has been made by modern human society to reduce the impact of human-generated waste through reducing and recycling. A potentially recyclable component of human-generated waste is carbon-based, such as ash or sludge from oil combustion or refining processes [1,2,3]. Waste carbon sources have the potential to be modified and functionalised in order to achieve desired characteristics in their future applications. This is due to the ability of carbon to exist in different allotropes, which are differentiated by their different bonds or structures, allowing them to possess different physical and electronic characteristics [4]. Therefore, carbon has the potential to be utilised for many electronic applications due to these promising characteristics. Typically, a main drawback of carbonaceous waste sources is the high concentration of toxic metallic impurities that must be removed prior to recycling [2]. However, even after the removal of such impurities, the purpose of this recycled carbon is restricted to conventional carbon black uses such as colouring agents or a rubber-related reinforcement material [5,6] and it sees limited use in electrical or electronic purposes. In this study, pre-treated, purified waste carbon black powder was subjected to laser annealing to alter the microstructure of carbon [7,8,9]. A pulsed femtosecond laser was used to selectively change the microstructure of waste carbon over other heat treatment methods, as it has been shown to induce a localised nanocrystallisation of carbonaceous bonds [10]. The heat-induced ablation caused by the femtosecond laser pulses have a minimal effect outside of the target area, to allow for a cleaner and higher resolution annealing of the surface of the target carbon powder [11]. Altering the electrical characteristics of the waste carbon would allow for a future investigation into waste carbon’s use in electronic applications.

## 2. Materials and Methods

**Figure 1:**
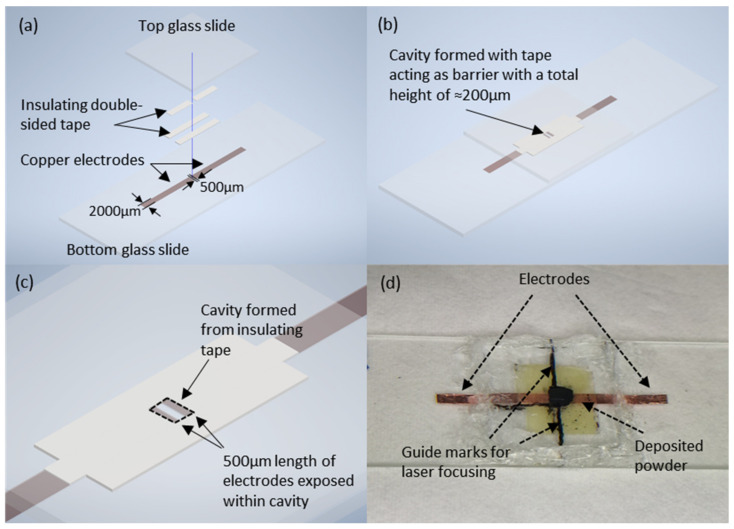
(**a**) Complete schematic of sample holder. (**b**) Schematic of sample holder with components placed together. (**c**) Magnified schematic drawing highlighting formation of cavity. (**d**) Optical image of sample with deposited carbon powder. Markings assist in the focusing of the laser.

Treated carbon black waste from the gasification of crude oil in refineries was prepared using methods described by P.W. Dong et al. [2] to remove its metallic impurities. Powder sample holders were made prior to laser annealing for the ease of handling. The design of the sample holder involved the placement of carbon powder within a cavity created using electrically insulating double-sided tape. Two copper tape electrodes spaced 500 µm apart were placed within the cavity to allow for the electrical I-V measurements. The waste carbon powder was then placed within the cavity, with the volume of powder controlled through filling the cavities with the powder followed by levelling off with a doctor blading technique. The samples were then enclosed by glass slides and sealed with silicone to provide an airtight seal, preventing oxidation whilst providing an additional adhesion mechanism for the structural integrity of the sample. The glass slides provide a transparent window for the laser power exposure to the powder in the experimental setting. The schematic and example of the samples is shown in Figure 1.

Femtosecond laser annealing was used as the heat treatment method to induce graphitisation in the waste carbon powder. A pulsed 800 nm laser with a pulse width of 140 fs and a pulse rate of 80 MHz from the Coherent Chameleon Ultra II (Santa Clara, CA, USA) system was used, with an average output power of 4 W and a peak pulse output power of >300 KW. The carbon powder samples were subjected to varying degrees of laser power using a polariser, attenuating the incident laser beam to the desired power level. The laser power used ranged from 0.104 W to 1.626 W with a gap of 5° polariser angle separation between each annealing power. The laser power was focused on the surface of the samples through a lens and traversed over the 500 µm gap between the copper electrodes to ensure that any changes in I-V characteristics were measured. The stage where the samples were placed moved at a fixed speed of 75 µm/s for all the samples to ensure that the output power was the only variable controlling the energy transfer of the laser energy onto the samples. The top of the carbon powder in the samples was marked to act as a focusing guide for the laser pre-annealing, to remove the focal point of the beam as a variable in the experiment.

## 3. Results and Discussion

**Figure 2:**
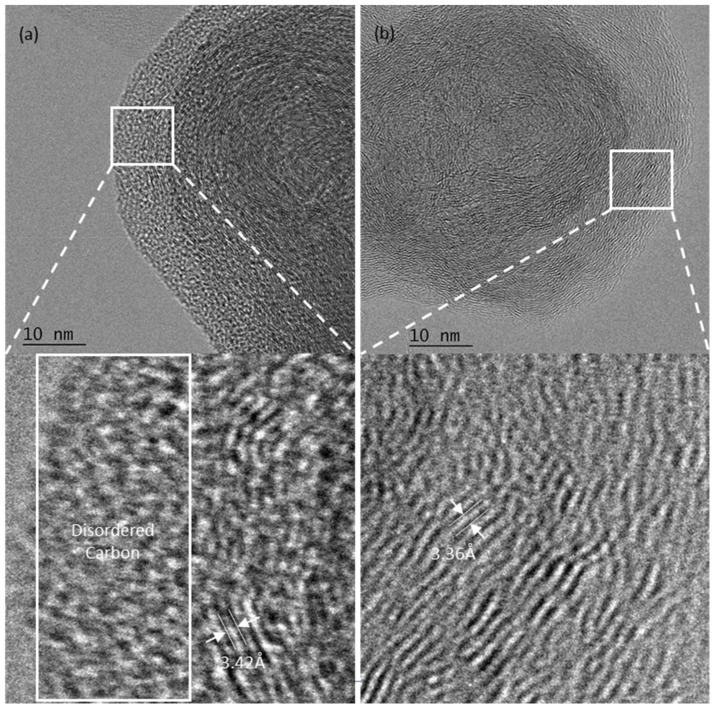
TEM images of carbon powder (**a**) pre-annealing and (**b**) post-annealing at 400,000× magnification.

Figure 2 shows the TEM images of the waste carbon powder taken pre- (Figure 2a) and post- (Figure 2b) laser annealing. The image of pre-annealed carbon particle shows a slight mix between amorphous and graphitic carbon, highlighted by the disordered region visible at the edge of the particle, while the image of post-annealed carbon shows a completely graphitic particle with no signs of disordered carbon present. The interlayer spacing of the carbon pre-annealing is found to be 3.42 Å, while the interlayer spacing of post-annealed carbon is 3.36 Å. This difference in interlayer spacing falls in line with the well-established interlayer spacings of disordered graphite (3.44 Å) and ordered graphite (3.354 Å), respectively [12]. This indicates the visible, structural impact of femtosecond laser annealing on treated carbon black waste.

**Figure 3:**
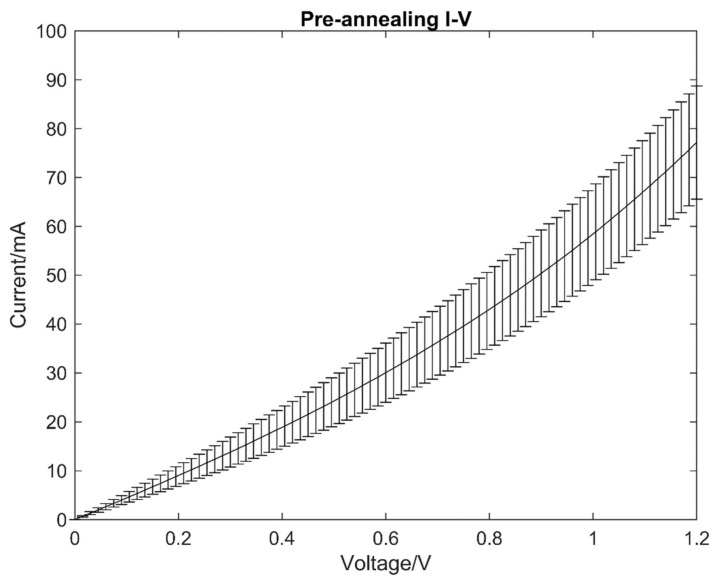
A representation of the I-V curve obtained from carbon powder samples.

The I-V characteristics of the carbon powder samples were taken pre- and post-annealing to be compared. The I-V characteristics were tested using a four-point probe station, and the average of the results with error margins for the samples pre-annealing are shown in Figure 3.

All I-V measurements were taken with forward and reverse voltage sweeps to 1.2 V at intervals of 0.015 V. The electrical conductivity of the copper electrodes was measured to be significantly larger than the carbon powder, and hence has been omitted in the measurement results. The results show the non-linear electrical conductivity of the carbon powder prior to laser annealing, which is a typical behaviour due to the amorphous nature of the carbon powder samples. Amorphous carbon conducts electricity through the free electrons offered by the sp^2^ hybridised atoms within the lattice. However, due to defects present in the molecular structure, the conduction between molecular groups within the lattice is limited to the low electric field regimes. Under higher electric fields, the localised charge carriers that would have otherwise recombined with the original lattice can migrate through the defects and conduct electricity [13,14,15]. This phenomenon can also be characterised as the Poole-Frenkel effect [13,16].

**Figure 4:**
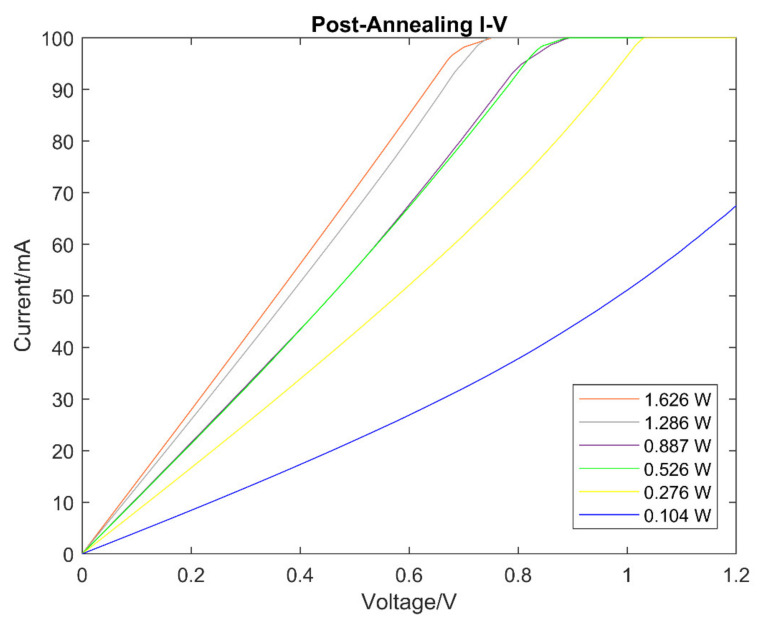
I-V curves of carbon powder samples post-annealing at different laser powers.

The I-V characteristics of the samples post-annealing are shown in Figure 4. The samples showed an increase in electrical conductivity, as shown with the increase in gradient of the I-V curves, at higher laser power levels. The increase in electrical conductivity is thus related to an increase in laser power. This result shows the proportional impact that increasing laser power has on the carbon powder, where an increased graphitisation in the carbon powder is observed with an increase in laser power. An observation was made for the laser power of 1.626 W; however, the high laser power may have caused a severe reduction in the carbon powder. Upon further inspection, all the samples above 0.526 W of laser power experienced some form of reduction, although the only observationally significant reduction was from the 1.626 W sample. Despite this point, the reduced amounts of annealed carbon powder became more conductive and can still conduct electrical current better than compared to pre-annealing. Conversely, there is a minimal observed change in electrical conductivity of less than 10% between pre-and post-annealing at lower laser powers (≤0.104 W).

**Figure 5:**
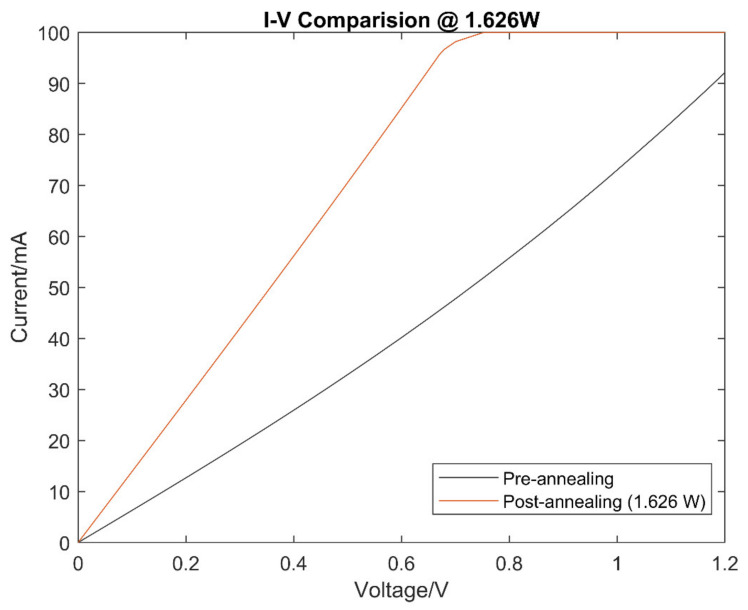
Pre- and post-annealing I-V characteristics of a sample exposed to 1.626 W of average laser power.

There was also an increase in linearity of the I-V characteristics with an increase in laser power levels. Figure 5 shows that the I-V curve of the carbon samples pre-annealing has a non-linear characteristic, and the I-V curve of the carbon samples post-annealing has changed to a linear I-V curve.

This suggests that it is possible to produce a more crystalline carbon structure from amorphous carbon through the process of laser annealing. Possible explanations of the results are that laser annealing altered the morphology of the carbon powder at higher laser powers. Graphitisation might have occurred at higher laser power samples, changing the amorphous carbon to a more crystalline-structured carbon, and thereby increasing their electrical conductivity, or correspondingly reducing their electrical resistances. The increased linearity in the I-V characteristics of post-annealed carbon suggests a reduction of disordered carbon within the structure by the heat induced from the laser annealing. With the correspondence between laser power and change in electrical characteristics, it can be concluded that the magnitude of laser annealing power has a direct impact on the changes induced to waste carbon powder.

Raman spectroscopy with a 532 nm laser was performed on the samples pre-and post-annealing to investigate, in detail, the graphitisation effect of laser annealing. The process where the ordered carbon within the structure of amorphous carbon increases is known as graphitisation, and conversely the increase in disordered carbon is characterised as amorphisation. The change in characteristics between crystalline graphite and amorphous carbon follows a gradual path known as the three-stage amorphisation trajectory [17], where the degree of graphitisation is characterised by the G peak position and the ratio of the D peak to G peak, or I(D)/I(G) ratio. Previous literature has found that an effective way to derive the peak positions and I(D)/I(G) ratios are by curve fitting the peaks using mathematical line shapes and evaluating the shapes for characterisation. The line shape used to map the D peak is the Lorentzian line function and the Breit-Wigner-Fano (BWF) line function for the G peak [18,19]. These line functions have been known to fit the Raman spectra of graphitic carbon without requiring additional functions. The three-stage model forms a benchmark to characterise the graphitisation state of tested carbon samples by relating the parameters to the actual structural makeup of the sample. The G peak position and I(D)/I(G) ratio of the Raman spectra can be used to provide an understanding on the graphitisation level of the carbon powder by comparing the parameters against the three-stage model. Changes identified from the differences of the Raman spectra pre- and post-annealing indicate graphitisation from laser annealing.

**Figure 6:**
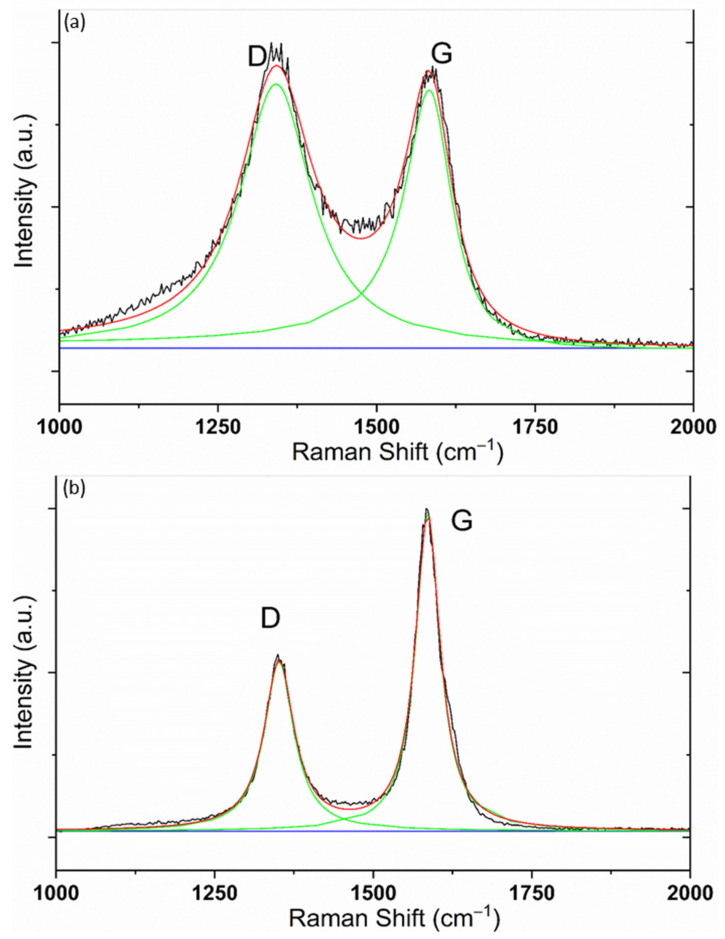
Raman curve fit for carbon samples (**a**) pre-annealing and (**b**) post-annealing at 1.626 W.

The I(D)/I(G) ratio can be obtained through the curve-fitting data, and the ratio gives a good insight of the changes in the microstructure of the carbon powder pre- and post-annealing. Figure 6 shows the curve-fitted Raman spectra for the samples pre-annealing (Figure 6a) and post-annealing at 1.626 W (Figure 6b). The first observation made from the Raman data was from the carbon sample annealed at a laser power of 1.626 W; This result corresponds to the reduction in the I(D)/I(G) ratio of the sample pre-annealing and post-annealing from 1.02 to 0.534, indicating that the carbon powder has transitioned from an amorphous-like microstructure to a graphite-like structure.

**Figure 7:**
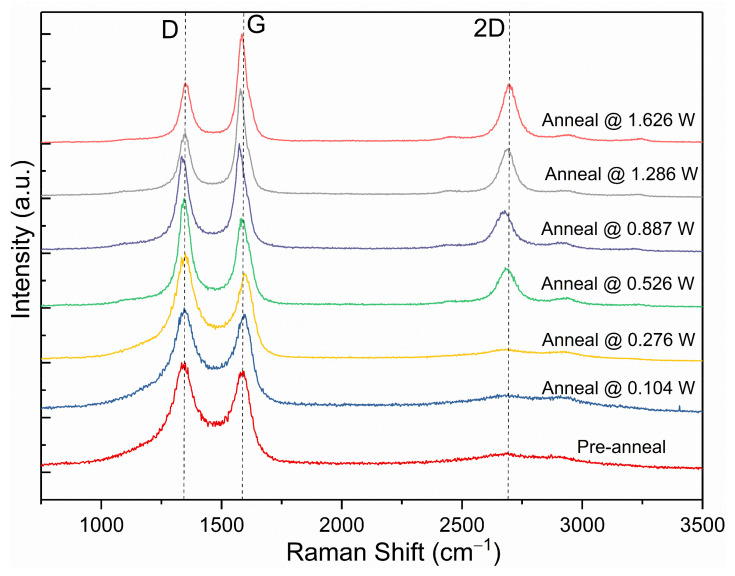
Raman spectroscopy comparison of carbon powder pre- and post-annealing at laser powers ranging from 0.104 W to 1.626 W.

The Raman spectra of the samples post-annealing at the tested laser powers were compared to pre-annealing in Figure 7. It was observed that at the laser power of 0.104 W, the carbon powder pre- and post-annealing have a similar I(D)/I(G) ratio with a minimal change of approximately 1%. These results indicate that for laser power below 0.104 W, the laser energy is too weak to induce any form of microstructural change onto the carbon powder. This is shown in the minimal change in the electrical resistance and Raman I(D)/I(G) ratio pre- and post-annealing.

At increasing laser powers, several observations with reference to the three-stage model indicate a microstructural change from amorphous-like carbon to graphite-like carbon. Firstly, I(D)/I(G) ratios migrated from 1.02 to 1.31 at 0.526 W before reducing to 0.534 at 1.626 W. The initial increase in I(D)/I(G) ratio can be attributed to the reduction in disordered sp^2^ bonds and corresponding increase in six-fold ordered sp^2^ bonds, increasing the intensity of the D peak in relation to the G peak as amorphous carbon is graphitised to nanocrystalline graphite. The further graphitisation of nanocrystalline graphite to bulk graphite results in the subsequent decrease in I(D)/I(G) ratio, as the D breathing mode is increasingly suppressed in increasingly ordered bulk graphite [17,18]. Secondly, the slight shift in G peak position from 1600 cm^−^^1^ to 1580 cm^−^^1^ at laser powers above 0.526 W further evidences the suppression of the D breathing mode, as the second D’ peak at ~1620 cm^−^^1^ is suppressed alongside the D peak [18]. Thirdly, the narrowing Full-Width-Half-Maximum (FWHM) of the G peak from 91.9 cm^−^^1^ to 47.4 cm^−^^1^ with increasing laser power is explained by the suppressing D′ peak, along with decreasing G peak dispersion in increasingly ordered graphite [18,20]. Finally, the appearance of the 2D peak at ~2700 cm^−^^1^ from 0.526 W evidences the presence of bulk graphite formed at the higher laser powers [20]. Thus, it can be deduced that as the waste carbon powder is subject to increasing levels of laser power, the microstructure of the carbon undergoes a greater magnitude of ordering to resemble bulk crystalline graphite. This result corresponds to the measured electrical I-V characteristics, as the ordered graphitic structure of the carbon post-annealing can provide an unobstructed electron flow, compared to the percolative electron flow in more disordered carbon.

## 4. Conclusions

The work presented in this article involves the use of femtosecond laser annealing to treat the carbon powder obtained from oil-refining waste. The laser annealing induces a localised graphitisation process, improving the electrical conductivity of the carbon powder to make it more suitable for future electrical applications.

From the experimental studies, the laser power required for a microstructural change was found to be between 0.526 W to 1.626 W. If the laser power is lower than 0.104 W, there is insufficient laser energy to induce discernible changes in the microstructure of the carbon powder. If the laser power is higher than 1.626 W, the carbon powder will be vaporised, resulting in very little carbon powder remaining. An intriguing point to note is that at the higher laser powers, there is still an increase in electrical conductivity of the carbon powder despite the vaporisation of the carbon powder. This indicates that only a very little amount of conductive carbon powder is required to produce an electrical conduction path. The ideal laser power for annealing has been determined to be in the range of 0.887 to 1.246 W, whereby the electrical conductivity has the greatest improvement, and the results correspond to a microstructural change from amorphous-like carbon to graphite-like carbon, as explained by the Raman data deduced with the three-stage model.

Therefore, there is potential for femtosecond laser annealing as a method to—selectively and locally—alter the structural and electrical characteristics of waste carbon powder. This will open the possibility for the functionalisation of waste carbon for use in future electronic applications.

## Data Availability

Not applicable.

## Abbreviations

FWHM	Full Width Half Maximum
